# Biases in COVID-19 Medical Resource Dilemmas

**DOI:** 10.3389/fpsyg.2021.687069

**Published:** 2021-08-12

**Authors:** Georgia Michailidou

**Affiliations:** Social Sciences Division, New York University Abu Dhabi, Abu Dhabi, United Arab Emirates

**Keywords:** allocation dilemma, ethical dilemma, gender bias, experiment, COVID-19

## Abstract

Accruing evidence suggest that COVID-19 is more fatal for males and minorities than other sub-populations. In this paper, we study medical dilemmas pertaining to the allocation of medical resources to evaluate whether existing social biases correspond to the demographic disparities of the pandemic. We develop and implement a choice experiment in which participants decide how to allocate scarce medical resources among COVID-19 patients with diverse demographic attributes. We find that participants violate optimal resource allocation significantly more often for the benefit of females. Males are almost half as likely to receive lifesaving resources even if these are medically more beneficial for them. We also find that participants are less likely to assign resources to patients with high compared to low income. Last, we find no evidence of patients' race affecting allocation preferences.

## Introduction

In less than one year from its appearance, COVID-19 claims millions of human casualties. In many regions, the spread of the virus was so rapid that the health care infrastructure was insufficient to grant everyone the intensive care they needed. In the months following February 2020, media and news extensively featured doctors and medical professionals reporting on the devastating situations taking place in emergency rooms and hospital corridors. There was no cure for COVID-19 and the resources were simply not enough to care for all the patients. Hard decisions had to be made (Robert et al., 2020; Shao, 2020; Truog et al., 2020). For navigating these decisions, many medical professionals reportedly opted for “the greatest amount of good for the greatest number” principle (Huang et al., 2019; Fink, 2020; Frakt, 2020; Mounk, 2020). With many moral dimensions to them, modern medicine has protocols for shorting patients' needs during pandemics and other medical emergencies (Verweij, 2009; Reid, 2020). These contemporary triages are explicit in that patients' gender, race, nationality, or other socioeconomic characteristics should have no bearing on doctors' assessments (Moskop and Iserson, 2007). For example, in the “Ethics and COVID-19” guidelines for doctors issued by WHO^1^, it is stated that “*Irrelevant characteristics of populations within countries, such as ethnicity, race or creed, should play no role in any resource allocation in any pandemic. This reflects our commitment to treating people with equal respect”*. The guidelines also emphasize that doctors follow, among others, the principle of “Utility” (best outcome) *to “justify the allocation of resources according to their capacity to do the most good”*, and the principle of “Prioritizing the worst off” *to justify the allocation of resources to those in greatest medical need or those most at risk*.^1^

The COVID-19, however, does not treat people with equal respect, i.e., does not ignore morally irrelevant factors. In the counts of the US National Center for Health Statistics^2^, male deaths outnumber female deaths by thousands, while Hispanics and Blacks' share of deaths is disproportionately higher than their respective share in the population^3^. Important evidence suggest that at least the gender inequalities are not local only to the USA (Jin et al., 2020; Peckham et al., 2021). These disparities in fatalities could be attributed to physiological or sociological factors or a combination of both. The scope of this study is to evaluate whether a set of social biases might produce experimental findings that overlap with the disparities of the pandemic. In more detail, we will be evaluating whether biases pertaining to gender, race, or income might be affecting the allocation of medical resources.

In particular, we use an experiment that allows us to evaluate whether there exist preferential biases in the hypothetical allocation of medical resources by examining whether participants violate the principles of utility and prioritizing the worse off for the benefit of a particular demographic. Indeed, we report that participants violate the said principles significantly more times for the benefit of a female than a male. When considering 913 dilemmas that involve allocations strictly between a male and a female, we observe that 240 patients that ought to have received the resource according its capacity to do the most good, did not. Out of those, 65.5% were males. That is, the number of males who “died” in our experiment due to biased resource allocation is 1.8 times that of females. Considering real deaths, Jin et al. (2020) report that the number of men who died from COVID-19 is 2.4 times that of women. Unlike the real COVID-19 death demographics, our results bring no evidence that patients' race affects allocation decisions. However, they reveal that patients' income does affect allocation decisions in a similar fashion gender does. Participants violate optimal allocation of resources for the benefit of low-income, compared to high-income patients. In a similar exercise, the number of high-income individuals who “die” due to biased allocation is 1.4 times that of low-income individuals.

In the shadow of COVID-19 and the current shortages in its vaccines, medical rationing dilemmas become ever so crucial. A strand of literature, methodologically closer to this paper, focuses on social aspects and human behavior during the pandemic. On the prevention front, experimental evidence suggests that mask wearing increases physical distancing (Seres, Balleyer, Cerutti, Friedrichsen, and Süer) and that men intend to wear face covering to a lesser extent than women do (Capraro and Barcelo, 2020). However, evidence of a fatalism effect are also reported (Akesson et al., 2020); the more infectious people believe that COVID-19 is, the less willing they are to take distancing measures. Further evidence show that risk, time and social preferences correlate with social compliance (Campos-Mercade et al., 2020; Müller and Holger, 2020) and that treatment-seeking behavior is affected by the perceived trustworthiness of the healthcare system (Antinyan et al., 2020). Evidence on intentions to vaccinate are documented to be sensitive to inconsistent risk messages from public health experts and elected officials (Thunstrom et al., 2020) while demand for antibody testing is found to be sensitive to price (Serra-Garcia and Szech, 2020). The study that we see as closer to this, in that it addresses the effect of demographics on COVID-19 related dilemmas, is the one by Huang et al. (2020). In that survey, participants allocate a one remaining ventilator either to an older patient who arrived at the hospital first or to a younger patient who arrived later. The authors report that when subjects employ a type of “veil of ignorance” reasoning, a method for deliberation that is supposed to reduce biases, they are more likely to allocate the ventilator to the younger patient. In this paper, using a different elicitation approach, one that is explicitly eliciting potential biases, we argue that patients' gender and economic status might be affecting allocation contemplations. Taken together the results of the two papers, albeit different in their objectives and methodologies, suggest that age, gender, and economic status might be factors that have a bearing in individuals' preferences for COVID-19 medical resources allocations.

## Methods

### Experimental Design

We conduct a choice experiment in which participants consider two COVID-19 related medical dilemmas, one associated with the principal of utility (best outcome) which we refer to as the Ventilator Dilemma (VD), and one associated with the prioritizing the worse off principle which we refer to as the Hospital Bed Dilemma (HBD). In the VD, two critically ill patients with COVID-19 would die unless they received a ventilator. There is only one available ventilator and participants decide which patient receives it. Life expectancy upon survival is the same between the two patients, 36 years, but one patient has 31% chance of survival if given the ventilator, the other 39%. Participants viewed vignettes that varied the following demographic characteristics of patients:

*Gender:* Participants view two patients who either have the same or different gender. Gender is conveyed by names and pronouns.

*Race/Ethnicity*: Participants view two patients who either have the same or different race. Race, either Black, or Latino, or White, is conveyed by names.

*Income:* Participants view two patients who either have the same or different income level. Income level, either $22,000, or $40,000, or $70,000 is explicitly stated in the vignettes.

*Parenthood:* Participants view either two patients who are not parents, or two patients who are both parents of two children. We did not allow for an allocation choice between a parent and a non-parent because we conjectured that, in these choice sets, minors' dependency on the parents might trigger behavioral patterns that we did not wish to study in this experiment. However, we opted for explicitly stating the parenthood status of patients to avoid participants' speculations about it and most importantly, to examine whether any biases arise only in the presence of children (for e.g., participants might be more willing to help mothers than fathers but not women over men when neither is a parent. However, none of our results changes when we control for the parenthood status).

In the HBD, two different patients exhibit mild symptoms that could be caused by COVID-19. If they remain in the hospital they will survive with certainty but if they are sent home, there is a chance their condition could worsen and they might die. There is only one hospital bed available and participants decide which patient receives it. Both patients have life expectancy of 41 years but the chance of survival if sent home is 74% for one patient and 67% for the other. Gender, race, income, and parenthood characteristics vary in the same way as in the VD.

In both dilemmas, participants had to allocated medical resources; in the first case, both patients are expected to live 36 years if they survive but their chances of survival differ. If the ventilator goes to the patient with the 31% chance of survival, the expected years of life saved amount to 11.16, and if it goes to the patient with the 39% chance of survival, the expected years of life saved amount to 14.04. Thus, the assignment of the ventilator to the patient with the lower chances deprives roughly 3 years of expected life from the other patient. Similarly, in the HBD, both patients are expected to live 41 years but their chances of survival differ. If untreated, one patient has 74% chance of surviving amounting to 30.03 expected years of life, and the other patient has 67% chance of survival amounting to 27.47 expected years of life. Thus, choosing to allocate the medical bed to the patient with the highest chances of survival deprives roughly 3 years of expected life from the other patient. According to the utility principle, in the VD the ventilator needs to be allocated to the patient with the 39% chance of survival, and in the HBD, the bed should be allocated to the patient with the 67% chance of survival.

Participants also took part in a belief elicitation exercise. Once they made their allocation decisions, they were asked to report how they thought 100 other participants from their respective counties behaved in the same two dilemmas but with different patients.

A Supplementary Video with the experiment as experienced participants is accompanying this submission and can also be viewed via this link^4^.

### Experimental Procedures

#### Choice Experiment

The procedural part of this study involved the recruitment of 1,842 individuals from the USA via Qualtrics, for a fixed fee. The study run from the 4th until the 13th of May 2020 (all dates before the death of George Floyd). The sample was balanced to be representative of USA population in the fields of gender, race, age (above 18), and parenthood (being a parent to at least one underage child). The participants had diverse educational and professional backgrounds and were not recruited as medical professionals. In the first part of the experiment, participants' basic demographics as well as exposure and attitudes toward COVID-19 were elicited. In the next part, participants reviewed the two medical dilemmas—one VD and one HBD each—. Finally, the belief elicitation was performed. Throughout the experiment, three thorough comprehension checks were performed. The 1,842 participants are those who passed the checks. We randomly varied patient characteristics across participants and their appearance as a left or right choice.

#### Name Check

A name check was performed prior to the choice experiment to verify whether the names presented in the vignettes conveyed gender and race accurately. 100 individuals from USA, recruited via Qualtrics on the 30th of April 2020, reviewed 31 names and guessed whether each belonged to a Black/Latino/White male or female. For the choice experiment, we used 24 of those names, four for each combination of race and gender. Each of the 24 names was guessed accurately by a minimum of 80% of participants. Attention and understanding checks were applied.

#### IRB and Preregistration

This research is under the NYUAD IRB Approval HRPP-2020-37 Social Science Online Games and Experiments. Consent was elicited according to the specifications of this approval and it occurred after informing participants of risks and benefits associated with participation. The choice experiment and its analysis, together with the name check were preregistered at as predicted #40175.

All data are accompanying this submission and can also be accessed via this link^5^.

## Results

**Figure 1:**
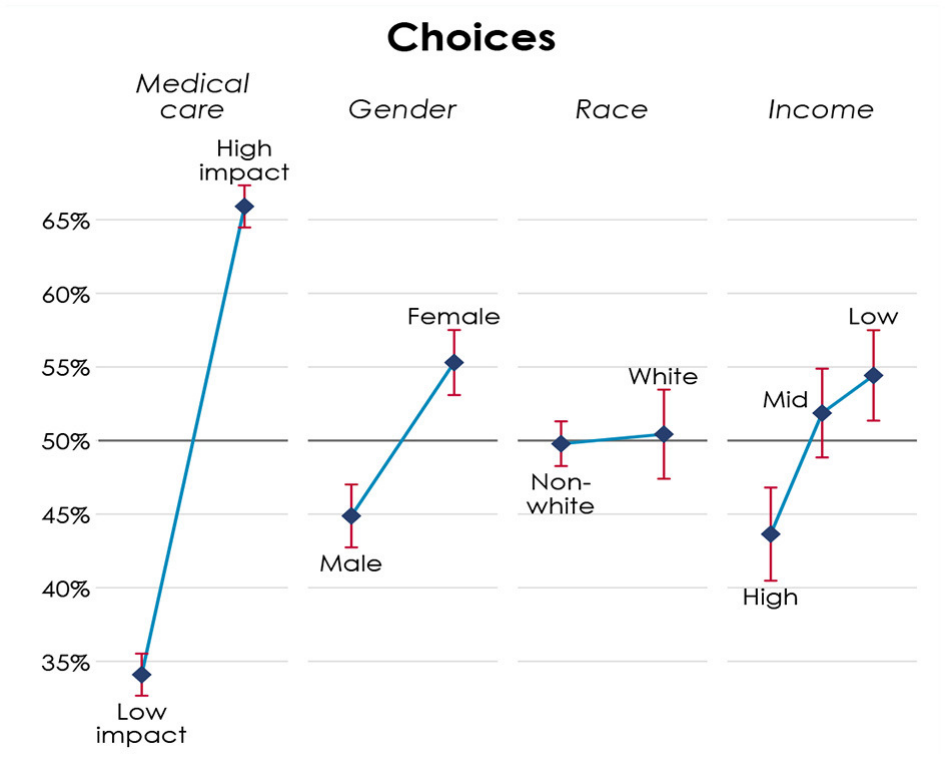
Allocation of medical resources across patients' attributes.

Turning to formal analysis, we consider the data via conditional logit models estimation. More specifically, we assume that a participant j who assigns medical assistance to a patient i in scenario k receives a psychological benefit (utility) given by:

$$\begin{array}{l} u_{jik}=\beta_{1}(SMed_{ik}--SNo_{ik})+\beta_{2}Female_{ik}+\beta_{3}Black_{ik}+\\ \;\beta_{4}Latino_{ik}+\beta_{5}IncomeLow_{ik}+\beta_{6}IncomeHigh_{ik}+\\ \;\Gamma Z_{ik}+\theta_{jk}+\varepsilon_{jik} \end{array}$$

where SMedik is the probability of survival with medical assistance of patient i in scenario k, SNoik is the probability of survival without medical assistance of patient i in scenario k, Femaleik is an indicator that patient i in scenario k is female, Blackik is an indicator that patient i in scenario k is black, Latinoik is an indicator that patient i in scenario k is latino, IncomeLowik is an indicator that patient i in scenario k has an income of $22k, IncomeHighik is an indicator that patient i in scenario k has an income of $70k, θjk corresponds to fixed effects for each participant-scenario combination, and εjik is a random variable capturing decision error. Participant j picks the patient i that gives the highest utility in scenario k. Figure 1 gives the graphical representation of each of these coefficients when we pool responses from both dilemmas.

Specifically, Figure 1 presents the estimated probability of choosing a patient, given their characteristics. Under medical care, High Impact stands for optimal^6^ choices according to the principles of utility and prioritizing the worse off. Participants apply these principles. They are 31.8 percentage points more likely to choose the patient that is consistent with these principles instead of the patient that is not, and this difference is highly significant (*p* < 0.01). Since patients' characteristics are randomly assigned, if there are no social biases, then the probability of choosing a patient with a given characteristic would be 50%. However, females are 10.4 percentage points more likely to be chosen than males, a difference that is also highly significant (*p* < 0.01). We do not find significant effects for race. Neither between low and medium income patients. However, participants are significantly less likely to assign resources to patients with high compared to low income (*p* < 0.01). The results do not change qualitatively if we consider the two dilemmas separately and they are not driven by any of the demographics of the sample. Participants do not behave differently when the patients have children and are not exhibiting any left or right choice bias.

**Figure 2:**
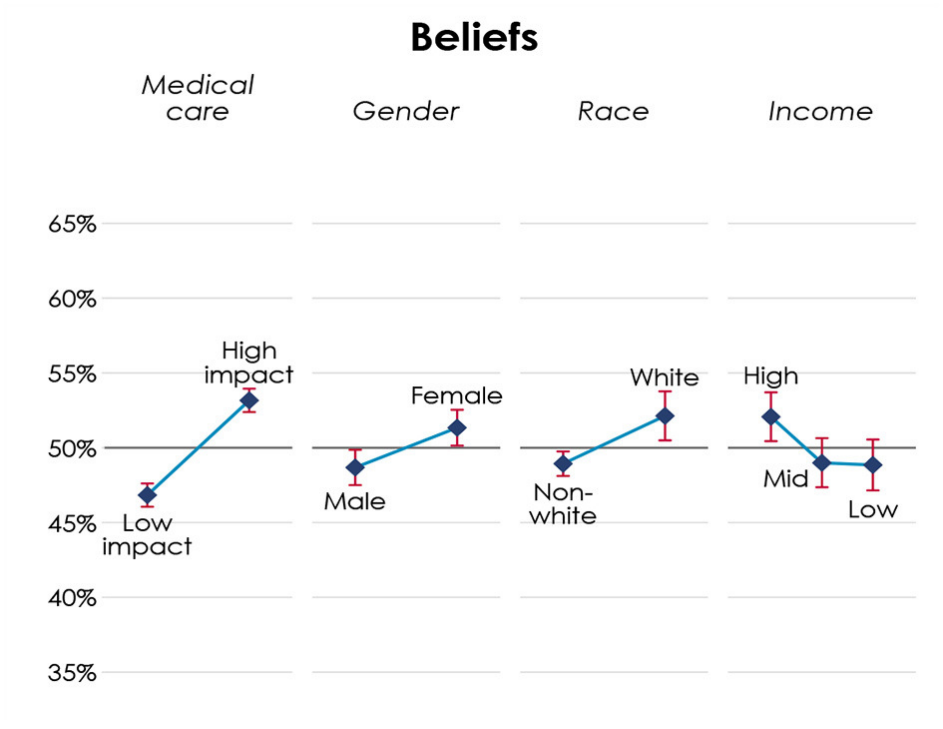
Beliefs over allocation of medical resources across patients' attributes.

Turning to the participants' beliefs about how 100 other participants from their respective county would behave in similar dilemmas, we apply analogous data estimation procedures and we summarize results in Figure 2 below.

Figure 2 presents both overlaps, and distinctions between choices and beliefs. Participants believe others are significantly more likely to allocate medical resources according to the principles of utility and prioritizing the worse off (*p* < 0.01), but the effect of principles in beliefs is smaller than in choices. Similarly, participants believe that others are significantly favor women over men (*p* < 0.05), albeit to a small degree than in choices. Unlike choices, participants believe others significantly favor white (*p* < 0.05) and high-income patients (*p* < 0.05).

## Discussion

Summarizing, we can infer that when allocating scarce medical resources, individuals significantly favor women and believe others favor women too. As briefly discussed, when isolating the dilemmas between males and females, this favoritism becomes more striking. As a proportion of all patient population considered here, males have 17.1% chances of not being allocated a ventilator when a ventilator would be more effective for them. The corresponding proportion for females is 9%. Either as a proportion of all patient population, or among those patients who should have been allocated the medical resource but didn't, males are almost half as likely to not receive the critical resource.

The overlap of choices and beliefs regarding this finding, hints to the existence of a behavioral norm. We hypothesize this norm could be associated with benevolent sexism. As proposed in Glick and Fiske (1996) and further discussed in Fiske (2018), benevolent sexism is a set of prosocial behaviors toward women, which are driven by and re-enforce stereotypical gender views such masculine dominance and feminine dependence. Related, there is evidence from various contexts on how females are more likely to elicit help and males to extend it (Eagly and Crowley, 1986; Sue, 2010) predominately motivated by the belief that, due to females' incompetence to lead any other role than a domestic one, males ought to “bear the burden of taking care of them” (Tajfel, 1969). Potentially, the pandemic brings forward society's protective instincts toward those it views as weaker and most vulnerable. Similarly to the practices of past centuries, during life threatening situations, we might still be guided by the “children and women first” code of practice, a collective behavior with various negative spillovers (Jost and Kay, 2005).

With regards to race, we do not find that participants over or under allocate medical resources to any of the race groups of our hypothetical patients; a finding that is not aligned with the significant over-representation of Hispanics and Blacks in COVID-19 deaths. However, in the belief elicitation exercise, participants guessed that Hispanics and Blacks were significantly less likely to be allocated the medical resources. We argue that this mismatch between choices and beliefs might be either due to erroneous perception over the prevalence of racism, or, due to concealed racism. In the first case, participants overestimate the extent to which Hispanics and Blacks might be experiencing disadvantageous discrimination. This mismatch between choices and beliefs might be due to participants overestimating the extent to which minorities experience discrimination or, due to participants showing less discrimination because of social desirability bias, yet projecting their racial biases when asked about the choices of others. These results are not at odds with the presence of benevolent sexism. While displaying racial discrimination comes with negative connotations, gender discrimination, concealed in the form of protectiveness, can resonate with past centuries' moral justifications. Although this study brings no support of contemporary racial bias, one should not exclude the possibility that other socio-economic and structural factors might be driving minorities' COVID-19 deaths over-representation; factors molded by years of well-documented institutional racism.

Benevolent and paternalistic attitudes might also explain the reasoning behind participants' preferences to favor low-income compared to high-income individuals. Similarly to women, low-income individuals might be seen as the weaker members of society that ought to be assisted at the expense of high-income individuals who might be assumed to have alternative means of assistance. Interestingly though, this preference does not seem to be a norm. Rather, it seems more as a curative counter to a perceived norm. When asked about their beliefs, participants think that others significantly favor high-income individuals, thus, when choosing, they opt to allocate resources to the low-income individuals, potentially to correct the perceived discrimination.

Given strong evidence that behavior elicited via the methodology we apply here is indicative of behavior in the real-world (Hainmueller et al., 2015), this paper brings significant evidence that COVID-19 medical resource allocation is socially biased in the domain of gender.

## Data Availability Statement

The original contributions presented in the study are included in the article/Supplementary Material, further inquiries can be directed to the corresponding author/s.

## Ethics Statement

The studies involving human participants were reviewed and approved by NYUAD IRB Approval HRPP-2020-37 Social Science Online Games and Experiments. The patients/participants provided their written informed consent to participate in this study.

## Author Contributions

GM conceptualized the study, designed the experiment, programmed the experiment, preregistered the experiment, executed the experiment, analyzed the data, and wrote the paper.

## Conflict of Interest

The author declares that the research was conducted in the absence of any commercial or financial relationships that could be construed as a potential conflict of interest.

## Publisher's Note

All claims expressed in this article are solely those of the authors and do not necessarily represent those of their affiliated organizations, or those of the publisher, the editors and the reviewers. Any product that may be evaluated in this article, or claim that may be made by its manufacturer, is not guaranteed or endorsed by the publisher.

## Footnotes

^1^ https://www.who.int/ethics/publications/ethics-covid-19-resource-allocation.pdf?ua=1

^2^Provisional death counts as of November, 2020.

^3^Considering the difference between COVID-19 weighted in relation to the geographic areas impacted by COVID-19 and adjusted for age differences across groups.

^4^ https://drive.google.com/file/d/1ooAdwdh9KbTX5Gxex11SPSp1_33TDHas/view

^5^ https://drive.google.com/file/d/1p3ERkV0af1OZ6NusNUTc-U4R5Ys4hHQb/view

^6^The principles of utility and prioritizing the worse off (as borrowed from WHO) are characterized here as optimal in the economics sense, meaning that not choosing according to these principles leads to the loss of 3 years of expected life.
